# Assessment of the *in Vitro* Antiprotozoal and Cytotoxic Potential of 20 Selected Medicinal Plants from the Island of Soqotra

**DOI:** 10.3390/molecules171214349

**Published:** 2012-12-03

**Authors:** Ramzi A. Mothana, Nawal M. Al-Musayeib, An Matheeussen, Paul Cos, Louis Maes

**Affiliations:** 1 Department of Pharmacognosy, College of Pharmacy, King Saud University, P.O. Box 2457, Riyadh 11451, Saudi Arabia; E-Mail: nalmusayeib@ksu.edu.sa; 2 Department of Pharmacognosy, Faculty of Pharmacy, Sana’a University, P.O. Box 33039, Sana’a, Yemen; 3 Laboratory for Microbiology, Parasitology and Hygiene (LMPH), Faculty of Pharmaceutical, Biomedical and Veterinary Sciences, Antwerp University, Universiteitsplein 1, 2610 Wilrijk-Antwerp, Belgium; E-Mails: an.matheeussen@ua.ac.be (A.M.); paul.cos@ua.ac.be (P.C.); louis.maes@ua.ac.be (L.M.)

**Keywords:** *in vitro*, antiprotozoal, antiplasmodial, antileishmanial, antitrypanosomal, medicinal plants, Soqotra

## Abstract

Malaria, leishmaniasis and human African trypanosomiasis continue to be major public health problems in need of new and more effective drugs. The aim of this study was to evaluate *in vitro* antiprotozoal activity of twenty endemic medicinal plants collected from the island of Soqotra in the Indian Ocean. The plant materials were extracted with methanol and tested for antiplasmodial activity against erythrocytic schizonts of *Plasmodium falciparum*, for antileishmanial activity against intracellular amastigotes of *Leishmania infantum* and for antitrypanosomal activity against intracellular amastigotes of *Trypanosoma cruzi* and free trypomastigotes of *T. brucei*. To assess selectivity, cytotoxicity was determined against MRC-5 fibroblasts. Selective activity was obtained for *Punica protopunica* against *Plasmodium* (IC_50_ 2.2 µg/mL) while *Eureiandra balfourii* and *Hypoestes pubescens* displayed activity against the three kinetoplastid parasites (IC_50_ < 10 µg/mL). *Acridocarpus socotranus* showed activity against *T. brucei* and *T. cruzi* (IC_50_ 3.5 and 8.4 µg/mL). *Ballochia atrovirgata*, *Dendrosicycos socotrana*, *Dracaena cinnabari* and *Euphorbia socotrana* displayed non-specific inhibition of the parasites related to high cytotoxicity.

## 1. Introduction

Human parasitic infections still represent a challenging public health problem worldwide, especially in tropical and subtropical regions. Malaria is the World’s most important parasitic disease, prevalent in about 100 countries and causing about 1 million deaths and 300 million infections per year [1,2]. Human African trypanosomiasis (sleeping sickness), Chagas disease and leishmaniasis affect the poorest populations in developing countries and have recently attracted the special focus of the WHO [3,4]. Leishmaniasis affects about 12 million people worldwide, with an increasing incidence of 2 million new cases every year and 350 million people at risk, despite the efforts made to fight the disease [5,6,7]. It is estimated that 300,000–500,000 individuals suffer from sleeping sickness in Sub-Saharan Africa and only 10–15% of the 60 million people living in risk areas are under surveillance [8,9]. In South America, 11–18 million people are infected by Chagas disease and 100,000 people are at risk of acquiring the disease [10]. Besides the lack of adequate efficacy, the small arsenal of available drugs for these neglected diseases is faced with side effects and the increasing problem of drug resistance [11], hence illustrating the urgent need for the development of cheap, safe and more effective drugs. Considering that 80% of the World population must still rely on traditional medicines using medicinal plants, less than 15% of all plant species have actually been investigated pharmacologically and/or phytochemically [12,13], which explains why a lot of recent research still focuses on traditional medicinal plants against protozoan diseases [14,15,16,17,18,19].

The island of Soqotra in Yemen has long been a land of mystery and is considered the “jewel” of biodiversity in the Arabian Sea. The long geological isolation and its fierce heat and many droughts have combined to create a unique and spectacular endemic flora. More than 800 plant species estimated to belong to more than 100 plant families occur of which 308 species are considered to be endemic [20,21]. Little has been reported on the pharmacology, biology and chemistry of medicinal plants from the island. In previous studies, we carried out investigations of some endemic plants from the island of Soqotra for antimicrobial, antiviral, enzyme-inhibitory and anticancer activity [22,23,24,25,26]. This study was carried out as a part of our continued exploration of medicinal plants from the Arabian Peninsula for antiplasmodial, antileishmanial and antitrypanosomal activity potential.

## 2. Results and Discussion

### 2.1. Results

A total of 20 plant species belonging to 16 families were collected from the island of Soqotra and submitted for *in vitro* screening in continuation of our previous studies on antiplasmodial, antileishmanial and antitrypanosomal activity of medicinal plants [27,28]. Table 1 lists the botanical names, specimen numbers, plant part used and the known traditional uses of the plants in the island of Soqotra. The results of the antiprotozoal activity are shown in Table 2.

**Table 1:** List of plants from the island of Soqotra that were screened in the study for antiprotozoal potential.

Plant species	Voucher specimen no.	Family	*Part extracted*	Traditional uses ^a^
*Acacia pennivenia* * Balf. f.	Mo-Sq28	Mimosaceae	L	As a paste around the breast for women with mastitis
*Acridocarpus socotranus* * Oliv.	Mo-Sq16	Malpighiaceae	L,S	Headaches, paralysis and muscle or tendon pain
*Aloe perryi* * Baker	Mo-Sq9	Aloaceae	R	Eye and stomach problems, constipation and malaria
*Ballochia atrovirgata* * Balf. f.	Mo-Sq15	Acanthaceae	S, L	Unknown
*Boswellia socotrana* * Balf. f.	Mo-Sq24	Burseraceae	B	Common cold, bronchitis, asthma and rheumatism
*Commiphora ornifolia* * J. B. Gillett	Mo-Sq23	Burseraceae	B	Antiseptic, diarrhea, dysentery and emesis
*Croton socotranus* * Balf. f.	Mo-Sq4	Euphorbiaceae	L, Fr	Wounds, anthelmintic
*Dendrosicyos socotrana* * Balf. f.	SP-A015	Cucurbitaceae	L, S	Urinary retention, cystitis, diabetes and problem with liver
*Dorstenia gigas* * Schweinf. ex Balf. f.	SP-M122	Moraceae	L, S	Flatulence, indigestion and skin diseases
*Dracaena cinnabari* * Balf. f.	SP-D225	Agavaceae	Re	Swellings, inflammations, sores, rashes, itching, stomach pain, haemostatic and oral health
*Euclea divinorum* Hiern	Mo-Sq1	Ebenaceae	R	For oral care, tooth ache, fungal diseases, sores, wounds and abscesses
*Euphorbia socotrana* * Balf. f.	Mo-Sq5	Euphorbiaceae	L	For skin diseases and wounds
*Eureiandra balfourii* * Cogn. & Balf. f.	Mo-Sq3	Cucurbitaceae	L	Unknown
*Hibiscus noli-tangere* * A.G.Mill.	Mo-Sq30	Malvaceae	L, R	For snake bite and fever in children
*Hypoestes pubescens* * Balf. f.	Mo-Sq12	Acanthaceae	L	Fungal skin diseases and scabies
*Lycium sokotranum* * Wagner & Vierh.	Mo-Sq20	Solanaceae	L, S	For stomach ailments and encourage the wound healing
*Maerua angolensis* DC.	Mo-Sq7	Capparaceae	L	To treat fever, aches and general malaise
*Punica protopunica* * Balf. f.	SP-D223	Punicaceae	Fr	Anthelmintic, peptic ulcers, dysentery, diarrhea, sores and wounds
*Rhus thyrsiflora* * Balf. f.	Mo-Sq18	Anacardiaceae	Fr, L	To treat anorexia, general tonic and for painful joints
*Teucrium sokotranum* * Vierh.	Mo-Sq22	Labiatae	Fl, L	As flavoring agent and for indigestion

* endemic plant, B: Bark, Fl: Flower, L: Leaves, R: Roots or rhizomes, Re: Resin, S: Stems, Fr: Fruits; ^a^ Most of the information of traditional use has been taken from [21] and native people.

**Table 2:** Antiprotozoal activity and cytotoxicity (IC_50_ µg/mL) of the methanolic extracts of the investigated plants.

Plant species	*P. falciparum*		*L. infantum*		*T. cruzi*		*T. brucei*		MRC-5
	IC_50_	SI	IC_50_	SI	IC_50_	SI	IC_50_	SI	IC_50_
*Acacia Pennivenia*	>64.0		>64.0		27.0 ± 4.7		8.3± 1.7	3.4	28.1 ± 2.6
*Acridocarpus socotranus*	21.6 ± 4.3		32.5 ± 6.2		8.4 ± 1.3	>7.6	3.5 ± 0.9	>18	>64.0
*Aloe perryi*	60.6 ± 7.8		>64.0		>64.0		31.7 ± 4.7		>64.0
*Ballochia atrovirgata*	2.9 ± 1.3	<1	6.0 ± 1.8	<1	0.6 ± 0.2	3.4	2.1 ± 0.8	1.0	2.1 ± 0.6
*Boswellia socotrana*	>64.0		50.8 ± 7.9		8.3 ± 2.4	3.9	9.3 ± 2.7	3.5	32.2 ± 5.9
*Commiphora parvifolia*	64.0		>64.0		>64.0		>64.0		>64.0
*Croton socotranus*	>64.0		>64.0		>64.0		>64.0		>64.0
*Dendrosicyos socotrana*	8.4 ± 2.1	<1	<0.25	<1	0.6 ± 0.1	1.1	7.3 ± 2.1	<1	0.7 ± 0.3
*Dorstenia gigas*	>64.0		>64.0		>64.0		40.3 ± 6.3		>64.0
*Dracaena cinnabari*	2.1 ± 0.9	3.6	8.1 ± 1.7	<1	4.1 ± 1.3	1.9	8.0 ± 1.8	<1	7.7 ± 2.0
*Euclea divinorum*	37.5 ± 4.7		>64.0		22.5 ± 4.7		33.1 ± 5.3		27.5 ± 3.6
*Euphorbia socotrana*	10.1 ± 1.8		7.5 ± 2.4	1.2	8.1 ± 1.6	1.1	1.9 ± 0.5	4.7	8.9 ± 1.2
*Eureiandra balfourii*	21.2 ± 2.9		6.0 ± 0.8	3.5	8.3 ± 1.3	2.5	8.1 ± 2.2	2.6	20.9 ± 2.8
*Hibiscus noli-tangere*	29.9 ± 4.7		32.5 ± 5.3		29.1 ± 4.6		8.2 ± 1.7	3.3	26.8 ± 3.8
*Hypoestes pubescens*	4.3 ± 3.1	7.6	7.5 ± 2.3	4.4	7.4 ± 2.1	4.4	2.0 ± 0.9	16.3	32.7 ± 4.2
*Lycium sokotranum*	35.2 ± 6.2		>64.0		23.8 ± 5.8		8.2 ± 2.2	2. 6	20.9 ± 2.6
*Maerua angolensis*	>64.0		>64.0		31.3 ± 6.3		33.7 ± 3.4		>64.0
*Punica protopunica*	2.2 ± 0.8	13.3	30.1 ± 6.8		32.9 ± 5.2		8.9 ± 1.9	3.3	29.5 ± 3.7
*Rhus thyrsiflora*	37.1 ± 4.9		>64.0		30.5 ± 4.3		34.0 ± 4.5		53.2 ± 9.3
*Teucrium sokotranum*	41.6 ± 6.3		>64.0		31.7± 6.1		7.9 ± 2.2	>8	>64.0
Chloroquine	0.3 ± 0.1	>213	-		-				>64.0
Miltefosine	-		3.32 ± 0.7	>19	-				>64.0
Benznidazole	-		-		2.2 ± 0.5	>29			>64.0
Suramin	-		-		-		0.03 ± 0.02	>2133	>64.0
Tamoxifen	-		-		-				11.0 ± 2.3

IC_50_ values of reference drugs are expressed in µM; SI: selectivity index (ratio of the IC_50_ value of the cytotoxicity against MRC-5 to the IC_50_ value of the antiparasitic activity): only listed for the extracts that complied with the activity criterion IC_50_ < 10 µg/mL.

#### 2.1.1. Cytotoxicity against MRC-5

Noticeable cytotoxic activity was shown by the methanol extract of *Ballochia atrovirgata*, *Dendrosicyos socotrana*, *Dracaena cinnabari* and *Euphorbia socotrana* (IC_50_ of 0.7–8.9 µg/mL). As such, the inhibitory activities observed in the different protozoan models must be regarded as non-specific and hence be considered without biological relevance (Table 2).

#### 2.1.2. Antiplasmodial Activity

Three plant extracts exhibited antiplasmodial potential (Table 2). *Hypoestes pubescens* and *Punica protopunica* displayed the highest potencies with high selectivity (IC_50_ 4.3 and 2.2 µg/mL, SI 7.6 and 13.3 respectively). The extract of *D. cinnabari* also showed a high antiplasmodial activity (IC_50_ of 2.1 µg/mL) but with low selectivity (SI 3.6). As indicated above, the extracts of *B. atrovirgata* and *D. socotrana* showed high inhibitory activity (IC_50_ 2.9 and 8.4 µg/mL), but are considered non-specific because of high cytotoxicity (IC_50_ 2.1 and 0.7 µg/mL).

#### 2.1.3. Antileishmanial Activity

Only two extracts demonstrated relevant activity against *L. infantum*, namely *Eureiandra balfourii* and *H. pubescens* (IC_50_ 6.0 and 7.5 µg/mL; SI 3.5 and 4.4 respectively) (Table 2). The inhibition observed for the extracts of *B. atrovirgata*, *D. socotrana*, *D. cinnabari* and *E. socotrana* is non-specific and linked to the high cytotoxicity against MRC-5 cells (IC_50_ < 10 µg/mL).

#### 2.1.4. Antitrypanosomal Activity

Eliminating the above mentioned cytotoxic plants, two extracts exhibited specific efficacy against *T. b. brucei*: *Acridocarpus socotranus* (IC_50_ 3.5 µg/mL; SI > 18) and *H. pubescens* (IC_50_ 2.0 µg/mL; SI 16.3) (Table 2). Both plant species were also active against *T. cruzi* (IC_50_ 8.4 and 7.4 µg/mL, respectively). Additionally, *T. cruzi* was also susceptible towards the extracts of *Boswellia socotrana* (IC_50_ 8.3 µg/mL) and *E. balfourii* (IC_50_ 8.3 µg/mL).

### 2.2. Discussion

The study of plants used in traditional medicine should continue to be seen as a rewarding strategy in the search for antiplasmodial, antileishmanial and antitrypanosomal drugs. Within our ongoing effort to identify novel drugs against malaria, human African trypanosomiasis, Chagas disease and leishmaniasis, this work focused on the rich flora of the island of Soqotra. It is important to point out that all 20 investigated plant species have never been evaluated before for antiprotozoal potential against *P. falciparum*, *L. infantum*, *T. brucei* and *T.* *cruzi*. Our results clearly show that the plant extracts displayed different levels of activity. Based on the activity (IC_50_) and selectivity, four plant extracts (*Acridocarpus socotranus*, *Boswellia socotrana*, *Hypoestes pubescens* and *Punia protopunica*) are considered as promising enough for further evaluation through bioguided purification and evaluation. Four other plants (*Ballochia atrovirgata*, *Dendrosicycos socotrana*, *Dracaena cinnabari* and *Euphorbia socotrana*) showed high cytotoxicity on MRC-5 cells and hence displayed non-specific inhibition of all tested protozoa.

Whereas the investigation of *Acridocarpus chloropterus* [29] showed no antitrypanosomal activity against *T. b. rhodesiense* STIB 900, the methanolic extract of the endemic *Acridocarpus socotranus* demonstrated in our screen a moderate activity with high SI against both *Trypanosoma* species. A previous phytochemical screening [26] showed the presence of terpenoids in this plant, which could be responsible for the observed activity. Almost similar results are obtained with *Boswellia socotrana* with moderate activity against both trypanosome species (IC_50_ < 10 µg/mL). These results are in agreement with literature data found for *Boswellia serrata* in which a cembrane-type diterpene (serratol) from the dichloromethane extract exhibited activity against *T. b. rhodesiense* [30]. The observed effect could be attributed to the cembrane-type diterpene compounds found in the volatile oil of the plant [31].

One of the more remarkable plants was *Hypoestes pubescens*, which demonstrated a pronounced and selective activity against all tested parasites. Similar antiplasmodial activity was found for *Hypoestes rosea*, a plant indigenous to Nigeria in which Ojo-Amaize *et al*. [32] reported the isolation of hypoestoxide, a diterpene that showed activity against different strains of cultured *P. falciparum*. In addition, several antifungal diterpenoids were isolated from *H. serpens* [33]. Our observed activities are likely correlated with the presence of the terpenoids and alkaloids, as found in a previous phytochemical screening [26].

*Punica protopunica* exhibited potent and selective antiplasmodial activity (IC_50_ 2.2 μg/mL), which is consistent with literature data of other *Punica* species. In earlier studies [34,35], high antiplasmodial and moderate antileishmanial activity was observed for *P. granatum* which was also confirmed in our previous study [28]. Reddy *et al*. [36] and Dell’Agli [37] attributed the antiplasmodial effect to the presence of phenolic compounds, including tannins such as ellagic acid, gallagic acid, punicalagins and punicalins that showed activity against *P. falciparum* D6 and W2 at IC_50_ values between 1.5 and 10 µM. Moreover, several studies reported that ellagic acid and some of its derivatives, e.g., flavellagic acid and coruleoellagic acid showed high *in vitro* activity against different *P. falciparum* strains irrespective their levels of chloroquine and mefloquine resistance. In additional, ellagic acid was shown to potentiate the activity of chloroquine, mefloquine, artesunate and atovaquone [38,39,40].

In an earlier study, pronounced antileishmanial and antiplasmodial activity was found for two species of *Dracaena,* namely *D. mannii* and *D. arborea*. The bioassay-guided fractionation led to the isolation of spiroconazole-A found to be responsible for the observed effect [41]. Several biflavonoids, homoisoflavanoids and flavonoids were isolated previously from *D. cinnabari* [42,43] and could contribute to the broad antiprotozoal effect observed in our study. Another cytotoxic plant is *Euphorbia socotrana* demonstrating non-specific activity. The genus *Euphorbia* has been the subject of abundant phytochemical and pharmacological exploration because of its potential medical applications. In contrast to our results, Maregesi *et al*. [44] reported antiplasmodial activity for the extract of *Euphorbia tirucalli*. The stilbene piceatannol was isolated from *Euphorbia lagascae* and screened against promastigotes of *L. donovani*, *L. infantum* and *L. major* with moderate activity [45]. Several triterpenoids isolated from *Euphorbia resinifera* and *Euphorbia officinarum* were found to inhibit *L. infantum* and *T. cruzi* [46].

## 3. Experimental

### 3.1. Plant Materials

The plants (Table 1) were collected from different locations of the island of Soqotra in the winter of 2008 and identified at the Soqotra Archipelago Conservation and Development Program (SCDP). Voucher specimens were deposited at the Pharmacognosy Department, Faculty of Pharmacy, Sana’a University. Table 1 lists the botanical name, voucher specimen, plant part screened and the reported medicinal uses of the plants.

### 3.2. Preparation of Extracts

The air-dried, powdered plant material (50 g) was placed in a Soxhlet apparatus and extracted with refluxing methanol (500 mL) for 8 h. The methanolic extract was then filtered off and concentrated using rotatory evaporator and freeze dried to remove any traces of methanol. The dried extracts were stored at −20 °C until used. Stock solutions for screening were prepared in 100% DMSO at 20 mg/mL.

### 3.3. Standard Drugs

For the different tests, appropriate reference drugs were used as positive control: tamoxifen for MRC-5, chloroquine for *P. falciparum*, miltefosine for *L. infantum*, benznidazole for *T. cruzi* and suramin for *T. b. brucei*. All reference drugs were either obtained from the fine chemical supplier Sigma-Aldrich (tamoxifen, suramin) or from WHO-TDR (chloroquine, miltefosine, benznidazole).

### 3.4. Biological Assays

The integrated panel of microbial screens and standard screening methodologies were adopted as previously described [47]. All assays were performed in triplicate at the Laboratory of Microbiology, Parasitology and Hygiene at the University of Antwerp (Belgium). Plant extracts were tested at 5 concentrations (64, 16, 4, 1 and 0.25 µg/mL) to establish a full dose-titration and determination of the IC_50_ (inhibitory concentration 50%). The final in-test concentration of DMSO did not exceed 0.5%. The selectivity antiprotozoal potential was assessed by simultaneous evaluation of cytotoxicity on a fibroblast (MRC-5) cell line. The criterion for activity was an IC_50_ < 10 µg/mL and a selectivity index (SI) of ≥4.

### 3.5. Antiplasmodial Activity

Chloroquine-resistant *P. falciparum* K 1-strain was cultured in human erythrocytes O^+^ at 37 °C under a low oxygen atmosphere (3% O_2_, 4% CO_2_, and 93% N_2_) in RPMI-1640, supplemented with 10% human serum. Infected human red blood cells (200 µL, 1% parasitaemia, 2% haematocrit) were added to each well and incubated for 72 h. After incubation, test plates were frozen at −20 °C. Parasite multiplication was measured using the Malstat assay, a colorimetric method based on the reduction of 3-acetyl pyridine adenine dinucleotide (APAD) by parasite-specific lactate-dehydrogenase (pLDH) [47,48].

### 3.6. Antileishmanial Activity

*L.infantum* MHOM/MA(BE)/67 amastigotes were collected from the spleen of an infected donor hamster and used to infect primary peritoneal mouse macrophages. To determine *in vitro* antileishmanial activity, 3 × 10^4^ macrophages were seeded in each well of a 96-well plate. After 2 days outgrowth, 5 × 10^5^ amastigotes/well, were added and incubated for 2 h at 37 °C. Pre-diluted plant extracts were subsequently added and the plates were further incubated for 5 days at 37 °C and 5% CO_2_. Parasite burdens (mean number of amastigotes/macrophage) were microscopically assessed after Giemsa staining on 500 cells, and expressed as a percentage of the blank controls without plant extract.

### 3.7. Antitrypanosomal Activity

*Trypanosoma brucei* Squib-427 strain (suramin-sensitive) was cultured at 37 °C and 5% CO_2_ in Hirumi-9 medium [49], supplemented with 10% fetal calf serum (FCS). About 1.5 × 10^4^ trypomastigotes/well were added to each well and parasite growth was assessed after 72 h at 37 °C by adding resazurin [50]. For Chagas disease, *T. cruzi* Tulahuen CL2 (benznidazole-sensitive) was maintained on MRC-5 cells in minimal essential medium (MEM) supplemented with 20 mM L-glutamine, 16.5 mM sodium hydrogen carbonate and 5% FCS. In the assay, 4 × 10^3^ MRC-5 cells and 4 × 10^4^ parasites were added to each well and after incubation at 37 °C for 7 days, parasite growth was assessed by adding the β-galactosidase substrate chlorophenol red β-D-galactopyranoside [51]. The color reaction was read at 540 nm after 4 h and absorbance values were expressed as a percentage of the blank controls.

### 3.8. Cytotoxicity against MRC-5 Cells

MRC-5 SV2 cells were cultivated in MEM, supplemented with L-glutamine (20 mM), 16.5 mM sodium hydrogen carbonate and 5% FCS. For the assay, 10^4^ MRC-5 cells/well were seeded onto the test plates containing the pre-diluted sample and incubated at 37 °C and 5% CO_2_ for 72 h. Cell viability was assessed fluorimetrically after 4 h of addition of resazurin. Fluorescence was measured (excitation 550 nm, emission 590 nm) and the results were expressed as % reduction in cell viability compared to control.

## 4. Conclusions

The antiprotozoal activity evaluation against *P. falciparum*, *L. infantum*, *T. brucei* and *T. cruzi* is being reported for the first time for twenty medicinal plants from the island Soqotra. *In vitro* studies confirm that the extracts of *Acridocarpus socotranus*, *Boswellia socotrana*, *Hypoestes pubescens* and *Punica protopunica* exhibited relevant antiplasmodial, antileishmanial or/and antitrypanosomal activity potential. Studies aimed at the isolation and structure elucidation of putative active constituents are in progress.
